# Shoulder Pain in COVID-19 Survivors Following Mechanical Ventilation

**DOI:** 10.3390/ijerph181910434

**Published:** 2021-10-03

**Authors:** Roberto Álvarez, María Fernanda del Valle, Pablo Cordero, Mariano del Sol, Pablo A. Lizana, Jorge Gutiérrez, Jorge Valenzuela, Rodrigo Muñoz-Cofre

**Affiliations:** 1 Servicio de Medicina Física y Rehabilitación, Hospital el Carmen, Maipú 9251521, Chile; roberto.alvarez18@gmail.com (R.Á.); fer.delvalle91@gmail.com (M.F.d.V.); pablo.cordero.v@gmail.com (P.C.); gutierrezdoc@gmail.com (J.G.); jorge.valenzuelav@redsalud.gob.cl (J.V.); 2 Escuela de Kinesiología, Universidad de Santiago de Chile, Santiago 9170022, Chile; 3 Centro de Excelencia en Estudios Morfológicos y Quirúrgicos, Universidad de La Frontera, Temuco 4811230, Chile; mariano.delsol@ufrontera.cl; 4 Laboratory of Morphological Sciences, Instituto de Biología, Pontificia Universidad Católica de Valparaíso, Valparaíso 2373223, Chile; pablo.lizana@pucv.cl; 5 Posdoctorado en Ciencias Morfológicas, Universidad de La Frontera, Temuco 4811230, Chile

**Keywords:** anatomy, pain, shoulder, COVID-19, peripheral nervous systems

## Abstract

COVID-19 has caused a certain proportion of patients to be hospitalized in intensive care units (ICU) and may cause musculoskeletal and neurological deficits following intubation and mechanical ventilation. The aim of this study was to quantify and describe the presence of shoulder pain in patients released from hospitals after suffering COVID-19. Patients with positive Apley tests were sent to a physiatrist for a clinical evaluation, ultrasound and electromyography (EMG). This evaluation was completed with a pain scale, joint range and shoulder muscle strength evaluations. Of the one-hundred-sixteen patients, seventy eight entered the respiratory rehabilitation program. Twenty patients were sent to the multidisciplinary shoulder team for positive Apley scratch tests. Of these twenty patients, one had only an EMG, ten had only ultrasounds, seven had an EMG and ultrasound and two did not need complementary tests. The twenty patients were sent to the physical therapist, with all presenting pain and diminished joint range and muscle strength in the affected shoulder. In this context, shoulder pain could be associated with the prone position in the ICU. We suggest time control and position change for patients on mechanical ventilation in a prone position with COVID-19.

## 1. Introduction

COVID-19 was first identified in Wuhan, China, in December 2019 and rapidly extended worldwide. By June 2021, there were already over 178 million cases diagnosed around the world [1]. It is estimated that around 5% of COVID-19 patients have severe symptoms, requiring care in highly complex units. Potential therapeutic measures for these patients can range from supplementary oxygen to mechanical ventilation (MV) in intensive care units (ICU) [2].

In the ICU, the prone decubitus (PD) position has been used to improve oxygenation. While the impact of the PD position is proven regarding increased oxygen (2), care must be taken when positioning patients since the pressure applied on specific body regions for prolonged time periods may cause nerve and muscle injuries, apart from the well-known risk of pressure ulcers [3].

Movement difficulties in the ICU due to the need to minimize health personnel exposure could increase joint rigidity risk [4]. Along with this, extrapulmonary manifestations of COVID-19, especially the immunological mechanisms, probably also contribute to the development of shoulder disorder development in patients with predispositions or backgrounds of rheumatic or traumatological pathologies [5]. Prior data from 254 Severe Acute Respiratory Syndrome (SARS-CoV-2) survivors showed that 53% of patients had joint pain, where the most common joints were the knee at 41% and the shoulder at 34% [4].

Some COVID-19 patients’ SARS progressed rapidly, needing prolonged ICU treatments. Some complications linked to these temporalities are polyneuropathy and myopathy, which compromise sensory and motor axons, respectively, and generally present as flaccid and symmetrical paralysis [5]. Apart from the presence of systemic neuropathies, focal neuropathies can be observed after prolonged ICU stays due to compression and/or traction of the brachial plexus or vascular structures [3].

Post-ICU syndrome covers certain complications throughout all areas of daily life following hospital discharge. On the physical side, the high rate of chronic musculoskeletal pain is notable [2]. In this regard, shoulder pain is frequent (5–80%), which can last for 6–12 months after leaving the hospital [6]. This affects upper limb function, generating higher costs to public health and the national economy, as well as lower productivity and other problems [2,3,4,5,6]. In addition, musculoskeletal disorders affect people’s quality of life, shoulder pain in particular [7,8]. The collateral problems associated with patients’ long mechanical ventilation stay could also be due to, or aggravated by, SARS-CoV-2 infection. Evidence indicates that SARS-CoV-2 infection affects the central nervous system as well as the peripheral nervous system, with consequences even several months after infection [9,10]. Hence, it is relevant to identify early shoulder problems in patients with shoulder pain. Therefore, the goal of this study was to quantify and describe the presence of shoulder pain in patients after their discharge following COVID-19 treatment.

## 2. Materials and Methods

### 2.1. Participants

In the present study, a cross-sectional design was used. All patients hospitalized in the ICU during the period of May–August 2020 were considered. After leaving hospital care, the patients enrolled in the post-COVID-19 cardiopulmonary rehabilitation program. The physical therapist in charge of the cardiorespiratory rehabilitation room entered all patients with positive Apley scratch tests [11] into the multidisciplinary upper limb team. The physiatrist carried out a clinical evaluation to indicate an upper limb electromyography (EMG) and/or an ultrasound of the glenohumeral joint, as needed. Subsequently, the physical therapist specializing in upper limbs performed functional shoulder evaluations, which is described in the Section 2.2. The inclusion criteria were: (i) patients hospitalized in the ICU connected to MV, over 18 years old and with COVID-19 diagnoses (confirmed by PCR (+) and/or thorax scanner compatible with COVID-19). The exclusion criteria were cognitive disorders impeding patients from following orders. This study was done in accordance with the Ethics Code of the World Medical Association (Helsinki Declaration) and had the approval of the Scientific Ethics Committee of the Central Metropolitan Health Service, Santiago, Chile (resolution 379/2021).

### 2.2. Measurements

From the electronic clinical records (Florence clinical version 19.3), we obtained sociodemographic data, medical backgrounds and clinical history, as well as days on MV and in prone position. Evaluations were divided in two: (i) The physical therapist from the respiratory rehabilitation program, as part of the entry evaluation, did an Apley scratch test [11]. This test has demonstrated a high level of inter-rater (0.89–0.97) and intra-rater (0.92–0.99) reliability [12]. (ii) Positive results led patients into the multidisciplinary upper-limb group, which did a clinical evaluation on upper limbs that was complemented with EMG and/or ultrasound. The following tests were done.

#### 2.2.1. Functional Mobility Assessment of the Shoulder

Since the loss of shoulder mobility in patients was important (inclusion criteria: Apley scratch test [11]), a modification of the shoulder mobility evaluation was performed, using three tests. In the anterior shoulder mobility test, the patient placed his or her hand on the contralateral shoulder. In the upper shoulder mobility test, the patient placed his hand on his occipital region. In the lower shoulder mobility test, the patient placed his hand on his upper lumbar region. In all three maneuvers, the patient had to be standing and could not perform any compensatory movements. The test was considered positive when the patient reached the indicated area (contralateral shoulder, occipital region, upper lumbar region, respectively).

#### 2.2.2. Shoulder Pain

A numerical rating scale was used. Patients were asked about their pain perception on a scale of 0 to 10, where 0 was the absence of pain and 10 was the worst pain imaginable. Patients were also asked to differentiate between pain while lying down and while performing daily activities [13].

#### 2.2.3. Range of Motion for Glenohumeral Joint Flexing and Abduction

To evaluate glenohumeral joint flexing and abduction, a universal goniometer was used (Patterson Medical, Bolingbrook, IL, USA). All measurements were performed by the same physiotherapist (RA). For both measurements, the patient was placed in a supine decubitus position with their hip and knee joints flexed, the soles of their feet on the bed and their hands and forearms in pronation. In the glenohumeral joint, the goniometer axis was located at the humeral head, with the fixed arm on the lateral line of the trunk aligned with the greater femoral trochanter, and the mobile arm in the median longitudinal line of the humerus in alignment with the lateral epicondyle of the humerus. Following this, the patient was asked to perform a maximum flexing of their shoulder to the point that it hurt. For the glenohumeral abduction, the goniometer axis was located in the anterior margin of the acromion, with the fixed arm on the anterior axillar line parallel to the sternum and the mobile arm on the longitudinal line of the humerus. Following this, the patient was asked to perform a maximal shoulder abduction to the point that it hurt [14].

#### 2.2.4. Shoulder Muscle Strength

To evaluate strength in the muscles used to perform abduction and flexing in the glenohumeral joint, we used the Daniels Scale which goes from 0 to 5. At 0, there is no muscular contraction. One presents palpable or visible contraction of the muscle group without movement. Two presents muscle contraction and the entire movement performed with assistance due to not being able to overcome gravitational force. At 3 the muscle can perform the movement, with gravity as the only resistance. At 4, the muscle contracts and performs the entire movement against gravity and moderate manual resistance. At 5, the muscle contracts and performs the entire movement against gravity and maximal manual resistance simultaneously [15].

#### 2.2.5. Palmar Pressure Strength

We used a hydraulic dynamometer (JAMAR^®^ Sammons Preston Inc., Salt Lake City, UT, USA). This test was done with the patient in a bipedal position, with their shoulder and forearm in a neutral position and their elbow at a 90-degree flex angle. The participant applied maximum pressure force for three seconds, making two attempts with a one-minute rest between each try, with the best value being selected [16].

#### 2.2.6. Upper Limb Nerve Conduction Study/Electromyography and Shoulder Ultrasound

These tests’ results were gathered from electronic clinical records (Florence clinical version 19.3).

### 2.3. Data Analysis

The data were tabulated and analyzed in the GraphPad Prism 5^®^ program. The results are presented as averages ± standard deviation or percentage.

## 3. Results

Out of 116 patients, 78 entered the respiratory rehabilitation program. Twenty patients were sent into the multidisciplinary shoulder team due to a positive Apley scratch test, of which seven were women and 13 were men, with an age of 63.16 ± 9.9 years. The average time for MV connection was 26.50 ± 17.8 days and 5.81 ± 2.9 days for PD position. A total of 70% of the sample had high blood pressure (systolic pressure ≥ 140 mmHg, diastolic pressure ≥ 90 mmHg), 15% had mellitus diabetes and 45% were obese (BMI ≥ 30 kg/m^2^). During MV, 100% of the sample were sedated and on neuromuscular blockers (Table 1).

Nine patients presented pain during repose, with the entire sample presenting pain during activity. Only one patient retained full joint range, while the other eight patients showed diminished glenohumeral joint flexing and abduction ranges. A total of 100% of the sample presented clinical alterations related to shoulder muscle strength and palmar pressure (Table 2).

Of the 20 patients, one received only an EMG, 10 only received ultrasounds, 7 had an EMG and an ultrasound and 2 of the patients needed no complementary tests (Table 3). Patients one and four had normal EMGs, although their ultrasounds showed structural shoulder disorders. Three patients had median nerve damage, three presented carpal-tunnel entrapment and one had polyneuropathy sensorimotor in the median and ulnar nerve. The ultrasounds mainly showed tendinosis and tendinopathies in the supraspinatus, subscapularis and infraspinatus muscles. Three patients presented signs suggesting frozen shoulder syndrome and another three indicated acromioclavicular arthrosis (Table 3).

## 4. Discussion

The principal results of this study indicate that patients referred for shoulder evaluation all presented pain during activity, with diminished joint range and shoulder muscle strength. These clinical limitations match the neurophysiological and/or structural disorders from the EMGs and ultrasounds, respectively. The mechanisms in nerve and muscle disorders within a COVID-19 context have not yet been clearly determined (1). Although there is little literature about shoulder pain in ICU patients suffering COVID-19-related pneumonia, recent results associate shoulder pain presence after leaving the ICU with various factors including age, previous pain, medical comorbidities and spending over 15 days in the ICU, which could be explained by diminished joint mobility and muscle mass loss [6].

Prolonged prone decubitus is used in MV patients to improve oxygenation during COVID-19-related pneumonia [2]. However, there are conditioning factors related to COVID-19 and ICU stays with repercussions on upper limbs. Researchers confer a certain “hypothetical capacity” of COVID-19 to act as a new pathogen which directly invades peripheral nerves and skeletal muscles via the ECA2 receptor [1]. Along with this, SARS CoV-2 also causes alterations in nervous and muscular microcirculation [3]. There are also anatomo-functional disorders linked to prolonged positioning during long ICU stays, which would generate nerve compression and rotator cuff pinching by increasing shoulder impingement, in contrast to the supine position [3,6].

The results of this study coincide with those of Puentes-Gutiérrez et al. (2020). This research group proposed to quantify shoulder pain presence two months after ICU discharge in patients hospitalized for SARS-CoV-2. In this way, out of 120 patients hospitalized in this unit, 38 received follow-up in the rehabilitation service following hospital discharge. Evaluations after two months showed that 18 people (47.4%) presented shoulder pain. The researchers attributed this pain to age, illness severity, prolonged opioid use, MV ≥ 12 days, ICU stay ≥ 15 days and the prone position [6]. Results from the present study coincide in age and BMI of the studied sample. However, there were differences in the number of days connected to MV, in prone decubitus position and corticoid use. Patients in this study spent more days on MV; all of them were in PD and received corticoid treatments. We suggest that the damages observed in both the EMG and ultrasounds could be linked to longer immobility in the PD position, together with these patients’ advanced age.

This study used an upper-limb EMG and a shoulder ultrasound, helping to locate the origin of the shoulder pain more precisely in the patients evaluated. Results indicated that four patients presented pain with a nerve origin, while another four had pain with both nerve and osteoarticular origins. This is similar to the findings of O’Sullivan et al. (2021) who described the location, severity and prevalence of upper limb nerve injuries linked to acute COVID-19 rehabilitation. Between March and June 2020, out of 256 patients entering the ICU, 15 patients with shoulder problems were selected. All patients with shoulder issues were on MV (32.5 days) and in PD position (7.3 cycles), as well as presenting significant muscle weakness and neuropathic pain, suggesting more proximal than distal nerve damage. Thirteen patients (87%) had multiple nerve injuries in their upper limbs. Most of them had injuries on the ulnar nerve, at the level of the cubital tunnel. The most common nerve injury site was the infraclavicular level, on the brachial plexus fascicles [3]. While there were similarities with the results of O’Sullivan et al., ICU days and PD position days were lower in the present study. This situation indicates that the cause of the upper limb nerve damage, apart from the time factor, is due to poor upper-limb positioning in PD.

In this context, positioning guides from the Faculty of Intensive Care Medicine (FCIM) suggest that the shoulder and elbow in the prone position should be at 80 and 90 degrees, respectively [17]. However, this position would generate stretching over the ulnar nerve at the elbow level. In this way, the safe pronation verification list from Oliveira et al. (2017) suggests positioning the patient with their elbow flexed at 70°, in order to prevent this damage. In recent months, guidelines have recommended an abduction of the glenohumeral joint under 70°, with the elbow bent at less than 70°. This prevents excessive abduction and lateral rotation positioning of the shoulder and therefore less brachial plexus traction [18]. Therefore, one of the strategies to prevent shoulder disorders is early movement, rotating upper limbs every 2–4 h and correct upper limb positioning [17]. We also suggest developing comparison guidelines to help improve PD positioning, along with sticking to stipulated timeframes.

Furthermore, the pharmacotherapy used for COVID-19 patients in ICUs has also been shown to cause physical aftereffects [3]. In patients on MV, using corticosteroids before removal is a strong predictor for muscle weakness (OR, 14.9; CI of 95%, 3.2–69.8; *p* < 0.001), regardless of dose or duration [19]. A prospective study which followed 203 ARDS survivors for a year also reported an inverse and significant association between corticosteroids (up to 40 mg/day of prednisone equivalent) and distance covered during the 6-min walking test (*p* = 0.032) [19]. These results indicate that glucocorticoids induce atrophy in rapid-contraction skeletal muscles, mainly due to their abundance of corticosteroid receptors [20]. In the evaluated sample, neuromuscular blockers were used on all 20 patients. These medications eliminate muscle tone and cause a decrease in dynamic containment in the shoulder, as well as place the glenohumeral joint at an inherent risk of subluxation in the PD position. This instability could therefore have contributed to generating the damage reported by the shoulder ultrasound [3,6].

The results from the clinical indicators used gave evidence of decreased movement range and muscle strength. In this regard, most of the disorders observed in the shoulder ultrasound indicated inflammation or breakage of the muscle group comprising the rotator cuff. Shoulder joint abduction biomechanics indicate that beyond 90° the decomposition of the force applied on the glenoid cavity tends to luxate it upwards and laterally, which makes rotator muscles contract against this luxation [21]. Along with this effect, the weight exercised by the scapular belt contributes to the excessive tension on the rotator muscle group. The extra load on the rotator muscles over time could therefore explain the damage seen in the shoulder ultrasounds [22].

This study has some limitations which must be clarified. (i) The number of participants was small. However, it coincides with the prevalence of recent studies [3,4]. (ii) Depending on the clinical evaluation, pertinent exams were indicated, which led to not all patients having ultrasounds and EMGs. (iii) In future research, the functional evaluation of the upper limb through surveys could complement the evaluations we performed. (iv) Although structural problems were shown by ultrasound, we cannot be certain if these issues began before hospitalization.

## 5. Conclusions

In summary: several patients discharged from the ICU presented shoulder pain, which needed to be identified with EMG and ultrasound tests. In this context, we suggest time control and position changing in prone COVID-19 patients on MV. We also recommend that earlier ultrasound examinations be considered in patients in the PD position on MV.

## Figures and Tables

**Table 1:** Anthropometric characteristics and morbid history of patients with shoulder pain, n = 20.

Variable	Mean ± SD
Gender (Male/Female)	7/13
Age (years)	63.16 ± 9.08
Weight (kg)	81.43 ± 11.82
Height (cm)	161.60 ± 15.82
BMI (kg/m^2^)	31.17 ± 6.81
Hypertension (n/%)	14/70
MD (n/%)	3/15
Obesity (n/%)	9/45
MV duration (days)	26.50 ± 17.78
PD duration (days)	5.81 ± 2.85
Corticosteroids (n/%)	18/90
Sedation (n/%)	20/100
Neuromuscular blockade (n/%)	20/100

SD: standard deviation; BMI: body mass index (≥30 kg/m^2^); hypertension (systolic blood pressure ≥ 140 mmHg and diastolic blood pressure ≥ 90 mmHg); MD: mellitus diabetes; MV: mechanical ventilation; PD: prone decubitus.

**Table 2:** Patient characteristics and shoulder evaluation results.

Patient	Gender	Age (Years)	Dominant Limb/ Painful Limb	Shoulder Pain (Points)		Range of Motion (Degrees)		Functional Mobility ^a^			SMS	PPS	
				Rest	Act	Flex	Abd	Ant	Sup	Post		R	L
1	F	70	right/right	3/10	7/10	90	85	Chin	Occipital	Gluteus maximus	M3−	5	5
2	F	59	right/left	5/10	8/10	160	120	Shoulder	Occipital	Lumbar	M3+	5	5
3	M	48	right/right	5/10	8/10	85	60	Shoulder	Occipital	Lumbar	M3−	10	10
4	F	63	right/right	0/10	4/10	160	110	Shoulder	Occipital	Lumbar	M3+	5	10
5	M	72	right/right	0/10	5/10	0	0	Shoulder	Umbilicus	Lumbar	M0	0	1
6	M	71	right/right	0/10	8/10	170	145	Neck	Shoulder	Gluteus maximus	M3	20	15
7	F	62	right/bilateral	0/10	6/10	160	140	Shoulder	Occipital	Lumbar	M3	10	10
8	M	53	right/left	0/10	5/10	90	90	Shoulder	Ear	Lumbar	M2+	28	28
9	F	63	right/left	4/10	8/10	120	95	Shoulder	Ear	Gluteus maximus	M2+	12	4
10	F	69	left/left	2/10	4/10	160	140	Shoulder	Occipital	Lumbar	M3−	5	5
11	M	41	right/left	5/10	4/10	170	160	Shoulder	Occipital	Lumbar	M3−	32	0
12	F	48	right/left	3/10	5/10	140	90	Shoulder	Ear	Lumbar	M3−	15	0
13	M	73	right/right	0/10	8/10	130	170	Shoulder	Occipital	Lumbar	M2+	10	2
14	M	63	right/bilateral	2/10	2/10	140	130	Shoulder	Occipital	Lumbar	M3	18	1
15	M	51	right/bilateral	3/10	5/10	180	180	Shoulder	Occipital	Lumbar	M3	42	30
16	M	54	right/left	0/10	7/10	110	70	Shoulder	Ear	Lumbar	M2+	5	10
17	M	51	right/right	0/10	6/10	140	100	Shoulder	Ear	Lumbar	M2+	18	15
18	M	62	right/right	0/10	4/10	145	125	Shoulder	Occipital	Dorsal	M3−	29	20
19	M	44	right/right	0/10	9/10	170	180	Shoulder	Occipital	Lumbar	M3+	20	19
20	M	51	right/left	0/10	5/10	100	90	Shoulder	Occipital	Gluteus maximus	M2+	45	35

M: male; F: female; SMS: Shoulder muscle strength; PPS: Palmar Pressure Strength (kilograms); Act: activity; Flex: flexion; Abd: abduction; Ant: anterior; Sup: superior; Post: posterior; R: Right; L: Left. ^a^ Functional mobility assessment of the shoulder.

**Table 3:** Results of the upper limb electrophysiological examination and shoulder ultrasound.

Patient	Electrophysiological Examination	Ultrasound
1	Normal	Degenerative rotator cuff pathology
2	-	Supraspinatus Tendinosis
3	Axonotmesis in right axillar nerve	Supraspinatus, subscapular and infraspinatus tendinopathy, and atrophy of muscles. Atrophied deltoid muscle
4	Normal	Acromioclavicular joint without significant pathological alterations. Signs suggesting frozen shoulder syndrome
5	Median and right ulnar nerves with sensorimotor axonal-type polyneuropathy. Right brachial plexopathy, severe in middle and lateral trunks	Tendinopathies in supraspinatus, subscapular and infraspinatus muscles with marked muscle atrophy, along with deltoid muscle
6	-	Complete thickness tear of supraspinatus tendon, signs of subacromial and subdeltoid bursitis. Signs of bicipital tenosynovitis and acromioclavicular arthrosis
7 *	-	-
8	Light median bilateral carpal tunnel level nerve entrapment. No myopathies or radiculopathies	Moderate supraspinatus tendinosis, without evidence of tearing
9	-	Supraspinatus tendinosis. Light subacromial and subdeltoid bursitis
10	-	Light subscapular and supraspinatus tendinosis. Light supraspinatus, subscapular and deltoid muscle atrophy
11	Median nerve entrapment at carpal tunnel level	-
12	-	Subacromial and subdeltoid bursitis
13	-	Subacromial and subdeltoid bursitis. Partial longitudinal tear of tendon from long portion of biceps. Calcific subscapular tendinitis. Supraspinatus tendinosis
14	-	Right shoulder: Supraspinatus tendinosis with anterior fiber tearing. Subacromial and subdeltoid bursitis. Left shoulder: Supraspinatus tendinosis. Subacromial and subdeltoid bursitis
15	-	Bilateral supraspinatus tendinosis. Tenosynovitis of long biceps head. Bilateral subacromial and subdeltoid bursitis. Degenerative changes in acromioclavicular joint
16 *	-	-
17	Sensory–motor polyneuropathy with sensory predominance.	Supraspinatus tendinosis with partial tearing. Tendinosis light in infraspinatus and in long tendon of the brachial biceps. Subacromial and subdeltoid bursitis. Degenerative changes in acromioclavicular joint
18	Median nerves with evidence of bilateral entrapment at carpal-tunnel level. No evidence of radiculopathies from C6 to C8-T1 bilaterally	Tenosynovitis of first extensor compartment with compromising of short thumb extensor. Thickening median nerve
19	-	Ultrasound study of right shoulder without pathological findings. Signs suggesting frozen shoulder syndrome
20	-	Supraspinatus tendinosis, without tearing evidence. Tissue with hyperemia adjacent to rotator tendons. Signs suggesting frozen shoulder syndrome

* Patients with clinical evaluation only.

## Data Availability

The datasets generated and/or analyzed during the current study are available from the corresponding author on reasonable request.
